# A case of vitamin B12 deficiency neurological syndrome in a young adult due to late-onset cobalamin C (CblC) deficiency: a diagnostic challenge

**DOI:** 10.11613/BM.2022.020802

**Published:** 2022-04-15

**Authors:** Scott Ailliet, Rik Vandenberghe, Toon Schiemsky, Lode Van Overbeke, Philippe Demaerel, Wouter Meersseman, David Cassiman, Pieter Vermeersch

**Affiliations:** 1 Clinical Department of Laboratory Medicine, University Hospitals Leuven, Leuven, Belgium; 2 Clinical Department of Neurology, University Hospitals Leuven, Leuven, Belgium; 3 Clinical Department of Laboratory Medicine, Ziekenhuis Oost-Limburg, Belgium; 4 Center of Metabolic Diseases, University Hospitals Leuven, Leuven, Belgium; 5 Clinical Department of Radiology, University Hospitals Leuven, Leuven, Belgium; 6 Department of Cardiovascular Sciences, KU Leuven, Leuven, Belgium

**Keywords:** vitamin B12, homocysteine, cobalamin C, subacute combined degeneration

## Abstract

Vitamin B12 deficiency can present with neurologic and psychiatric symptoms without macrocytic anaemia. We describe a case of late-onset cobalamin C deficiency which typically presents with normal serum vitamin B12 concentrations, posing an additional diagnostic challenge. A 23-year-old woman with decreased muscle strength and hallucinations was diagnosed with ‘catatonic depression’ and admitted to a residential mental health facility. She was referred to our hospital for further investigation 3 months later. Heteroanamnesis revealed that the symptoms had been evolving progressively over several months. Magnetic resonance imaging (MRI) of the brain showed diffuse symmetrical white matter lesions in both hemispheres. Routine laboratory tests including vitamin B12 and folic acid were normal except for a slight normocytic, normochromic anaemia. Over the next 6 weeks her symptoms deteriorated, and she became unresponsive to stimuli. A new MRI scan showed progression of the white matter lesions. The neurologist requested plasma homocysteine (Hcys) which was more than 8 times the upper limit of normal. Further testing revealed increased methylmalonic acid and the patient was diagnosed with adult-onset cobalamin C deficiency. This case illustrates that Hcys and/or methylmalonic acid should be determined in patients presenting with neuropsychiatric symptoms suggestive of vitamin B12 deficiency with a normal serum vitamin B12 to rule out a late-onset cobalamin C deficiency.

## Introduction

Vitamin B12, also known as cobalamin (Cbl), is a water-soluble micronutrient that is synthesized only by microorganisms. Dietary sources include milk, eggs, fish, and meat. Vitamin B12 is necessary for DNA synthesis and methylation reactions. Vitamin B12 deficiency can present with a range of hematologic, cardiovascular, neuropsychiatric, and gastrointestinal symptoms (*1*).

Vitamin B12 deficiency is caused by inadequate intake, malabsorption, chemical inactivation, or an inborn error of the cobalamin metabolism (*1*). Cobalamin malabsorption due to pernicious anaemia is the most frequent cause of vitamin B12 deficiency in patients aged 60 or older but is typically not observed below the age of 30 (*2*). In young adults, in contrast, the most frequent causes of vitamin B12 deficiency are strict vegan diet, atrophic gastritis, medications that reduce gastric acid (*e.g.,* proton pump inhibitors), recreational nitrous oxide use, and disorders affecting intestinal absorption such as inflammatory bowel disease or celiac disease.

Serum B12 concentration is the first-line test in adults suspected of vitamin B12 deficiency. In general, a value well below the limit of the reference interval is indicative of probable vitamin B12 deficiency, whereas a value well above this limit is considered indicative of sufficient B12 status (*1*). In patients with low normal or only moderately decreased vitamin B12 additional measurement of homocysteine (Hcys) or methylmalonic acid (MMA) is recommended to rule out functional B12 deficiency (*3*).

Inborn errors of the cobalamin metabolism are rare. Combined methylmalonic aciduria and homocystinuria due to an inborn deficiency of cobalamin C (CblC) is the most common inborn error of cobalamin metabolism with an estimated prevalence of 1:200,000 births. The age of onset varies from prenatal to adult, but most patients present in the first year of life. More than 160 published cases of late-onset CblC deficiency (onset > 12 months) have been published (Pubmed search 26th November 2021) including more than 30 cases of adolescent-onset (12-17 years) and more than 30 cases of adult-onset (≥ 18 years) (*4*-*7*). The clinical presentation differs from the infantile presentation (*6*, *8*). Late-onset CblC deficiency is mainly dominated by neurological and neuropsychiatric symptoms. Renal thrombotic microangiopathy, haemolytic uremic syndrome, pulmonary hypertension and pulmonary thrombotic events have also occasionally been described in adult-onset CblC deficiency (*9*, *10*). Adult patients with an inborn error of the intracellular cobalamin metabolism C deficiency (CblC) typically present with normal to high-normal serum vitamin B12 concentrations and without macrocytic anaemia, posing a diagnostic challenge (*2*, *8*).

We present a case of late-onset CblC deficiency in a 23-year-old woman with neuropsychiatric symptoms in whom the diagnosis was initially missed due to a high normal vitamin B12.

## Case report

In June 2014 a 23-year-old woman who experienced severely decreased mobility, decreased muscle strength and hallucinations was diagnosed with ‘catatonic depression’ by a psychiatrist and admitted to a residential mental health facility. Her medical history included surgery and chemotherapy for an Ewing sarcoma in 2002 and 2003 when she was eleven years old. In 2012, at the age of 21, she had a transient period of abnormal gait and incontinence, but fully recovered after rehabilitation. Brain magnetic resonance imaging (MRI) at that time showed diffuse white matter lesions which were ascribed to the chemotherapy treatment 9 years before.

Her family insisted she was not depressed, and she was referred to our hospital for further investigation in September 2014 three months after her admission. On neurological examination her speech was impaired and her responses to questions were inadequate. Eye movements could not be tested due to lack of cooperation. Hetero anamnesis revealed that the symptoms had been evolving progressively over several months. Brain MRI showed extensive diffuse white matter lesions. Ophthalmological evaluation with fundoscopy showed no retinal or optic disc abnormalities. Routine laboratory tests including aspartate aminotransferase (AST), alanine aminostransferase (ALT), and creatinine (Cobas c702, Roche Diagnostics, Basel, Switzerland) at that time were normal except for a mild normocytic, normochromic anaemia with a haemoglobin value of 116 g/L (reference interval (RI): 120-160 g/L) (Table 1) (XE-5000, Sysmex Europe, Norderstedt, Germany). Serum vitamin B12 was 448 pmol/L (RI: 141-489 pmol/L) and folic acid in red blood cells was 3235 nmol/L (RI: 1185-2848 nmol/L) (Cobas Modular E, Roche Diagnostics, Basel, Switzerland). Investigations for infections of the central nervous system (bacterial, mycobacterial, viral, fungal) were negative. Isoelectric focusing and IgG index for detection of intrathecal IgG synthesis also did not reveal any abnormalities.

**Table 1:** Laboratory test results of the index case at the time of diagnosis and her siblings

	**23 y, female** **homozygous** **(index case)**	**28 y, female** **homozygous**	**36 y, female** **heterozygous**	**39 y, female** **heterozygous**	**38y, female** **no mutation**	**34 y, female** **no mutation**	**RI**
Vitamine B12 (pmol/L)	448	537	/	/	/	/	141–489
Hcys (µmol/L)	101.5	78.3	10.1	4.2	12	7.9	6.0–13.0
MMA (mmol/mol Crea)	802	41	1	< 1	< 1	< 1	≤ 1
MCA (mmol/mol Crea)	18	2	< 1	< 1	< 1	< 1	≤ 2
Hb (g/L)	116	130	/	/	/	/	120–160
RBC (x 10^12^/L)	3.67	4.42	/	/	/	/	3.90–5.60
MCV (fL)	89.4	88.7	/	/	/	/	76.0–69.0
AST (U/L)	24	/	/	/	/	/	≤ 32
ALT (U/L)	31	/	/	/	/	/	≤ 31
Creatinine (µmol/L)	46.9	/	/	/	/	/	45.1–84.0
Crea – creatinine. Hcys – homocysteine. Hb – haemoglobin. MCA – methylcitric acid. MCV – mean red blood cell volume. MMA – methylmalonic acid. RBC – red blood cell count. AST – aspartate aminotransferase. ALT – alanine aminotransferase. y – years. RI – reference interval.							

**Figure 1:**
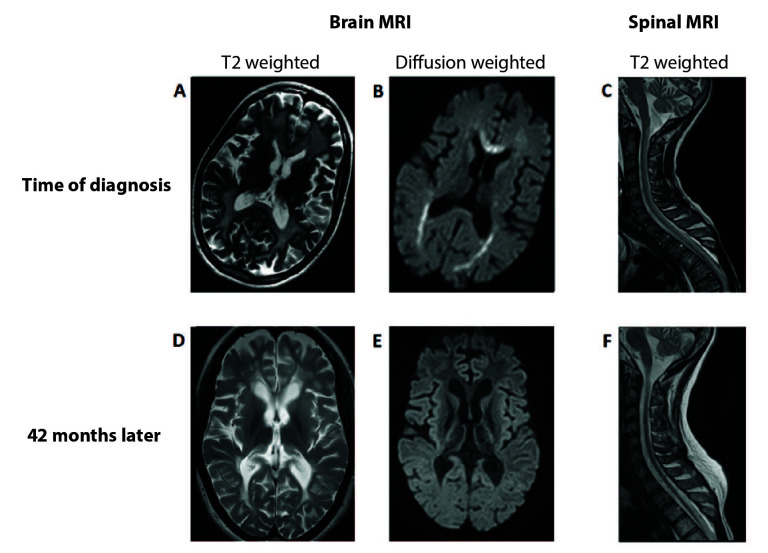
Magnetic resonance imaging (MRI) of the brain and spinal cord. At the time of diagnosis, brain MRI showed diffuse white matter lesions in both hemispheres (T2) with diffusion restriction confirming the acute nature of the lesions (Figure 1A and 1B), and spinal MRI revealed subacute combined degeneration of the cord (Figure 1C). After 42 months of treatment, brain MRI showed cerebral atrophy and a regression of the white matter lesions (Figure 1D and 1E) and spinal MRI showed regression of the spinal lesions (Figure 1F).

Over the next 6 weeks, her symptoms deteriorated. She lost her speech and did not respond anymore to visual and tactile stimuli. A second brain MRI revealed new diffusion restrictive foci in the genu of the *corpus callosum* and in the optic radiation on both sides (Figure 1). Spinal cord MRI revealed diffuse involvement of the dorsal spinal column (Figure 1). Given the rapidly progressive deterioration, the neurologist requested plasma Hcys to exclude a possible functional vitamin B12 deficiency despite the high normal plasma vitamin B12. Homocysteine measured with ACL TOP 500 CTS (Instrumentation Laboratory Company, Bedford, USA) was more than 8 times the upper limit of RI (101.5 µmol/L (RI: 6.0-13.0 µmol/L)). The suspected diagnosis of a vitamin B12 deficiency was further supported by an increased MMA, 802 mmol/mol creatinine (≤ 1 mmol/mol creatinine) and 2-methylcitric acid (MCA, 18 mmol/mol creatinine (≤ 2 mmol/mol creatinine)) on urine organic acid analysis with gas chromatography mass spectrometry Thermo Scientific Trace GC and ITQ mass spectrometer (Interscience, Louvain-la-Neuve, Belgium) (Table 1). Based on the organic acid results, an inborn error of the cobalamin metabolism was suspected.

## Treatment and outcome

Treatment with a daily dose of 25 mg hydroxocobalamin administered subcutaneously was started immediately. Within 3 weeks the patient started to slowly recover and plasma Hcys (15.6 µmol/L), urinary MMA (23 mmol/mol creatinine) and MCA (5 mmol/mol creatinine) almost normalized. At the latest follow up consultation to date, her neuropsychiatric symptoms had disappeared. She had regained speech, was communicative and oriented. Motor and visual functions were still recovering, but she remained severely impaired without ambulation, with limited use of her arms and hands, she still suffered from urinary incontinence and had limited vision. A follow-up MRI in 2018, four years after diagnosis, showed partial regression of the white matter lesions with signs of cerebral atrophy (Figure 1). The spinal cord MRI revealed a regression of the dorsal spinal column involvement (Figure 1).

The suspected diagnosis of CblC deficiency was confirmed by decreased total [^57^Co] uptake (8.7 pg/mg protein (RI: 40-156 pg/mg protein) and decreased production of methylcobalamin (15% of total uptake (40-76%)) and adenosylcobalamin (4.3% of total uptake (14-28%)) in cultured fibroblasts, compatible with a CblC or CblD deficiency (*11*). The diagnosis was further confirmed by genomic amplification and direct sequencing of all coding exons of *MMACHC*, including the flanking regions, which identified a homozygous mutation c.566G>A in exon 4 of the *MMACHC* gene (*12*). Familial screening revealed that the parents were consanguineous, and one sister had an increased plasma Hcys and urinary MMA (Table 1). She was started on pre-emptive treatment with 25 mg hydroxocobalamin administered subcutaneously three times *per* week, leading to normalization of plasma Hcys and urinary MMA and MCA. She was also homozygous for the mutation c.566G>A in exon 4 of the *MMACHC* gene. Two of the other four sisters were heterozygous and two sisters did not carry the mutation (Table 1). The index case and her sister who were diagnosed with CblC deficiency signed an informed consent.

## Discussion

This case underlines the challenge to diagnose late-onset CblC deficiency which typically presents with normal to high-normal serum vitamin B12 concentrations and without macrocytic anaemia (*2*, *8*). It was only due to the alertness of the neurologist, who requested plasma Hcys of the rapid progressive deterioration, that the patient was diagnosed with late-onset CblC deficiency.

**Figure 2:**
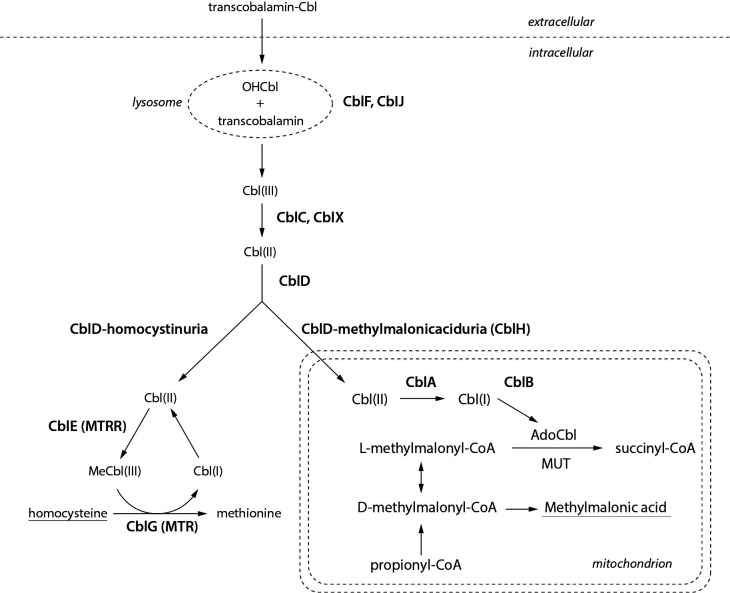
Intracellular metabolism of cobalamin including the location of the defect for the complementation groups (CblA to CblJ and CblX). The proposed valence state of the cobalt atom of cobalamin is shown in parentheses. AdoCbl – adenosylcobalamin. Cbl – cobalamin. MeCbl – methylcobalamin. MTR – methionine synthase. MTRR – methionine synthase reductase. MUT – methylmalonyl-CoA mutase. OHCbl – hydroxocobalamin.

There are several inborn errors that disrupt intracellular cobalamin metabolism which are classified as complementation groups CblA to CblJ and CblX (Figure 2). They have overlapping clinical manifestations and can result in isolated methylmalonic academia (CblA, CblB, and CblD variant 2, mut), combined methylmalonic academia with hyperhomocysteinaemia (CblC, CblD, CblF, and CblJ, CblX, cblX-like), or isolated hyperhomocysteinaemia (CblD variant 1, CblE and CblG). Deficiency of CblC is the most common inborn error of vitamin B12 metabolism with an estimated incidence of 1:100,000 to 1:200,000 births (*13*).

Serum vitamin B12 currently remains the first-line test in patients with suspected cobalamin deficiency but normal serum vitamin B12 does not always indicate sufficient concentrations at the cellular level. There are also a number of causes of analytical interference which can lead to a false-normal result including monoclonal gammopathy, anti-intrinsic factor antibodies and high-dose biotin supplements. There were no arguments to suspect an analytical interference in our patient (normal protein electrophoresis, no anti-intrinsic factor antibodies, and no biotin supplements).

There is currently no ‘gold standard’ test for the diagnosis of cobalamin deficiency. Early clinical markers of cobalamin deficiency, occurring even in the presence of normal serum cobalamin concentrations, are increased serum or urine MMA and plasma Hcys concentrations. Elevation of plasma Hcys and MMA distinguishes vitamin B12-deficient from folate-deficient patients which have normal MMA. Methylmalonic acid and Hcys are recommended as second-line tests when serum vitamin B12 concentrations are normal in patients with a high suspicion of B12 deficiency (*14*). However, there are some key drawbacks to consider in the application of these second-line tests: availability of these tests is limited, definitive cut-off points to define clinical and subclinical vitamin B12 deficiency states are not available, given the variety of methodologies used, and a lack of available local reference ranges.

Neuropsychiatric manifestations associated with acquired vitamin B12 deficiency include motor, sensory and autonomic symptoms, cognitive impairment, mood, and psychotic symptoms. The most common neurologic findings in vitamin B12 deficiency are peripheral neuropathy, numbness, and ataxia. Peripheral neuropathy has a wide range of differential diagnoses but in young adults it is a red flag for potential inherited neuropathy (Charcot Marie Tooth), toxic exposure or vitamin deficiency (*15*-*17*). The subacute combined degeneration of the posterior and lateral columns is a symptom of severe vitamin B12 deficiency. It manifests as spastic paraparesis, impaired proprioception and vibratory sensation, and the presence of Babinski’s reflex and a positive Romberg’s sign upon neurological examination. Optic neuropathy symptoms include scotomas and symmetric, progressive visual loss. Epilepsy can occur. Radiologic aspects of vitamin B12 deficiency include periventricular leukoencephalopathy, seen as white matter lesions on CT scan. Brain MRI typically shows nonspecific leukoencephalopathy, ranging from isolated periventricular white matter lesions to diffuse white matter loss on T2 weighted MRI. Spinal cord lesions can be seen as dorsal column involvement on MRI, with the “inverted V sign” being indicative of subacute combined degeneration of the spinal cord (*2*, *18*).

Patients with CblC deficiency with adolescent- or adult-onset have predominant neurologic and neuropsychiatric manifestations and typically a normal serum vitamin B12 as was the case in our patient (*4*, *8*, *10*). Additional measurement of Hcys in plasma, MMA in plasma or MMA in urine is required in an adult patient presenting with neurologic or neuropsychiatric symptoms with a normal serum vitamin B12 to exclude a CblC deficiency. Cbl A, CblB or CblD (variant 2) deficiency could be missed if only Hcys is measured, but we did not find any reports in the literature of late onset presentation (≥ 12 years). If only MMA is measured, a CblE, CblD (variant 1) or CblG deficiency could be missed. We did not find any reported cases of late onset (≥ 12 years) CblE or CblD (variant 1) deficiency and one case of CblG deficiency with onset of neuropsychiatric symptoms at the age of 21.

The observation that the clinical situation of the patient started to deteriorate after she was admitted to a residential mental health facility suggests that a sudden change in lifestyle with reduced vitamin B12 uptake (*e.g.* diet) while she was admitted for a prolonged period may have precipitated her symptoms. The fact that the patient and her sister are homozygous for the c.566G>A mutation in the *MMACHC* and were asymptomatic until adulthood suggests this mutation is associated with significant residual function of the *MMACHC* protein. The heterogenic presentation of CblC deficiency in general may be attributed to differences in the stability of the mRNA or residual function of *MMACHC*. Preferential allele transcript expression and the overall amount of *MMACHC* transcript may also contribute to genotype-phenotype correlations and differences in age of onset (*19*).

There are a number of limitations to our case report. First, Hcys and MMA were not measured during the initial diagnostic work-up because these tests are not reimbursed in Belgium for the diagnosis of acquired vitamin B12 deficiency. Second, we did not measure holotranscobalamin as this test was not yet routinely available in Belgium in 2014. Holotranscobalamin may, however, be normal in patients with inborn errors of intracellular Cbl metabolism just like serum vitamin B12 (*20*).

## Conclusion

Cobalamin C deficiency should be considered in the differential diagnosis of adult patients with progressive neuropsychiatric symptoms. Adult CblC deficient patients can present with symptoms typical of B12 deficiency with normal serum B12 concentrations. We propose to measure MMA in serum, MMA in urine and total Hcys in plasma when confronted with patients presenting with neurological symptoms typical of B12 deficiency despite normal serum vitamin B12 concentrations.
